# Interplay between driveline infection, vessel wall inflammation, cerebrovascular events and mortality in patients with left ventricular assist device

**DOI:** 10.1038/s41598-023-45110-6

**Published:** 2023-10-29

**Authors:** Juliane Hupe, Hans Worthmann, Kim K. Ravenberg, Gerrit M. Grosse, Johanna Ernst, Axel Haverich, Frank M. Bengel, Karin Weissenborn, Jan D. Schmitto, Jasmin S. Hanke, Thorsten Derlin, Maria M. Gabriel

**Affiliations:** 1 Department of Neurology, Hannover Medical School, Carl-Neuberg-Str. 1, 30625 Hannover, Germany; 2 Department of Cardiac, Thoracic, Transplantation and Vascular Surgery, Hannover Medical School, Hannover, Germany; 3 Department of Nuclear Medicine, Hannover Medical School, Hannover, Germany

**Keywords:** Cardiac device therapy, Molecular imaging, Chronic inflammation

## Abstract

In patients with left ventricular assist device (LVAD), infections and thrombotic events represent severe complications. We investigated device-specific local and systemic inflammation and its impact on cerebrovascular events (CVE) and mortality. In 118 LVAD patients referred for ^18^F-FDG-PET/CT, metabolic activity of LVAD components, thoracic aortic wall, lymphoid and hematopoietic organs, was quantified and correlated with clinical characteristics, laboratory findings, and outcome. Driveline infection was detected in 92/118 (78%) patients by ^18^F-FDG-PET/CT. Activity at the driveline entry site was associated with increased signals in aortic wall (r = 0.32, *p* < 0.001), spleen (r = 0.20, *p* = 0.03) and bone marrow (r = 0.20, *p* = 0.03), indicating systemic interactions. Multivariable analysis revealed independent associations of aortic wall activity with activity of spleen (β = 0.43, 95% CI 0.18–0.68, *p* < 0.001) and driveline entry site (β = 0.04, 95% CI 0.01–0.06, *p* = 0.001). Twenty-two (19%) patients suffered CVE after PET/CT. In a binary logistic regression analysis metabolic activity at the driveline entry site missed the level of significance as an influencing factor for CVE after adjusting for anticoagulation (OR = 1.16, 95% CI 1–1.33, *p* = 0.05). Metabolic activity of the subcutaneous driveline (OR = 1.13, 95% CI 1.02–1.24, *p* = 0.016) emerged as independent risk factor for mortality. Molecular imaging revealed systemic inflammatory interplay between thoracic aorta, hematopoietic organs, and infected device components in LVAD patients, the latter predicting CVE and mortality.

## Introduction

Due to an increasing incidence of heart failure^1^ and the shortage of donor organs for transplantation, the use of left ventricular assist devices (LVADs) has become an essential treatment option for patients with end-stage heart failure^2^. However, even in the era of a new LVAD generation, adverse events occur frequently^3,4^ and play a major role in hospitalisation, morbidity, and mortality^1,5^. These complications include infections, bleeding events and ischemic or haemorrhagic strokes—the latter being associated with permanent disability and a lower chance of heart transplantation^6,7^.

LVAD-specific infections—mostly due to infection of the driveline^8,9^—occur within the first year after implantation in up to 27% of LVAD patients^9,10^. Early diagnosis is of significant relevance^11^ for timely antibiotic therapy or even surgical revision^12^. Recent evidence suggests that infections of wounds, bloodstream or drivelines are involved in stroke pathogenesis in LVAD patients^8,9^. Survival appears to be lower in patients with infection-associated strokes compared with those suffering stroke without infection^11^.

Clinical molecular imaging using ^18^F-fluorodeoxyglucose (FDG)-positron emission tomography/computed tomography (^18^F-FDG-PET/CT) directly visualizes inflammatory activity, allowing early diagnosis, accurate assessment of the extent of infections and prognostic information on outcome^13,14^. Importantly, ^18^F-FDG-PET/CT may also characterize systemic inflammation crosstalk based on metabolic activity of lymphoid and hematopoietic organs, in particular the spleen, lymph nodes and bone marrow^15–17^. In addition, ^18^F-FDG-PET/CT is a validated method for imaging inflammatory activity in the atherosclerotic vessel wall^18–20^, which has been associated with cardiovascular events^19^. Systemic inflammation is a response of the organism to an infectious or non-infectious agent, characterized by a generalized release of proinflammatory cytokines with chronic activation of the immune system. Of importance, this does not necessarily imply a clinically overt systemic inflammatory response syndrome (SIRS) complicated by the failure of one or more organs, but rather a generalized reaction to an infectious or non-infectious inflammatory stimulus^21^.

Based on these preclinical and clinical observations, we aimed to further characterize the inflammatory interplay between LVAD components, arterial inflammation, lymphoid and hematopoietic organs to determine its impact on cerebrovascular events and mortality in a large cohort of LVAD patients.

## Materials and methods

### Study group and clinical data

We retrospectively reviewed the records of 411 patients who underwent LVAD implantation at an academic tertiary LVAD center between January 2015 and March 2021. A total of 121 patients with ^18^F-FDG PET/CT were identified, three of whom were excluded due to advanced metastatic carcinosis as competing reason for thrombogenicity and stroke.

^18^F-FDG-PET/CT was performed to specify an unclear focus of infection or infected LVAD components (n = 107), to validate for heart transplantation (n = 8) and to accomplish tumor staging (n = 3). At least one further ^18^F-FDG PET/CT was performed in 57 patients (2 PET/CTs n = 30, 3 PET/CTs n = 20, > 4 PET/CTs n = 7; in total n = 211), providing data for longitudinal analysis.

Medical history before and after LVAD implantation and clinical data in temporal proximity to PET/CT were assessed including mean arterial pressure (MAP) and diagnosis of driveline infection (DLI). In accordance with the in-house proceeding, driveline entry sites are screened at every outpatient presentation or as a daily routine in hospitalized patients by specialized infection control nurses. The screening includes a laboratory check, an examination of the driveline entry site by specialized infection control nurses and a microbiological smear of the driveline entry site. Out-patients are routinely seen every 3 months. If a superficial DLI is diagnosed at this regular check-up based on the standardized criteria mentioned below, empirical antibiotic therapy is initiated, and close wound management is conducted in the outpatient clinic. If the DLI does not regress under antibiotic therapy, or if there are signs of a deep DLI with potential need of surgical care according to ISHLT^22^ or a systemic infection, the indication for a PET/CT examination is made. Clinically apparent DLI was assessed using the consensus-based standardization of definitions in LVAD infections of the International Society for Heart and Lung Transplantation (ISHLT). DLI was diagnosed in the presence of a positive aseptic microbiological smear at the driveline entry site or in case of missing microbiological smear in combination with a) local hyperthermia, b) erythema and c) purulent wound secretion^22^. Complete blood count (CBC) including white blood cell count (WBC), C-reactive protein (CRP) and estimated glomerular filtration rate (eGFR) at the time of PET/CT were collected. Regular presentation of all patients to the center’s outpatient clinic allowed collection of clinical follow-up data and assessment of adverse events from hospital records and imaging data. Cerebrovascular events (CVE) and mortality after PET/CT were recorded with a median follow up interval of 1105 (IQR 598–1723) days. The minimum observation period was 116 days after PET/CT.

The study was approved by the local ethics committee of Hannover Medical School, Germany (Approval No. 9114_BO_S_2020) and was conducted in accordance with the relevant guidelines and regulations. The study was conducted in accordance with the Declaration of Helsinki. All patients or their legal guardians gave informed consent to pseudonymized data processing and publication for scientific research purposes.

### PET/CT imaging and image analysis

Whole-body ^18^F-FDG PET/CT was performed 60 min post injection using a Biograph mCT Flow 128 scanner (Siemens Healthineers, Erlangen, Germany). As previously described^13^, PET/CT was performed after injection of a median of 304.5 MBq of ^18^F-FDG after a minimum of 4–6 h fasting. Low-dose CT was conducted for anatomical correlation and attenuation correction.

**Figure 1:**
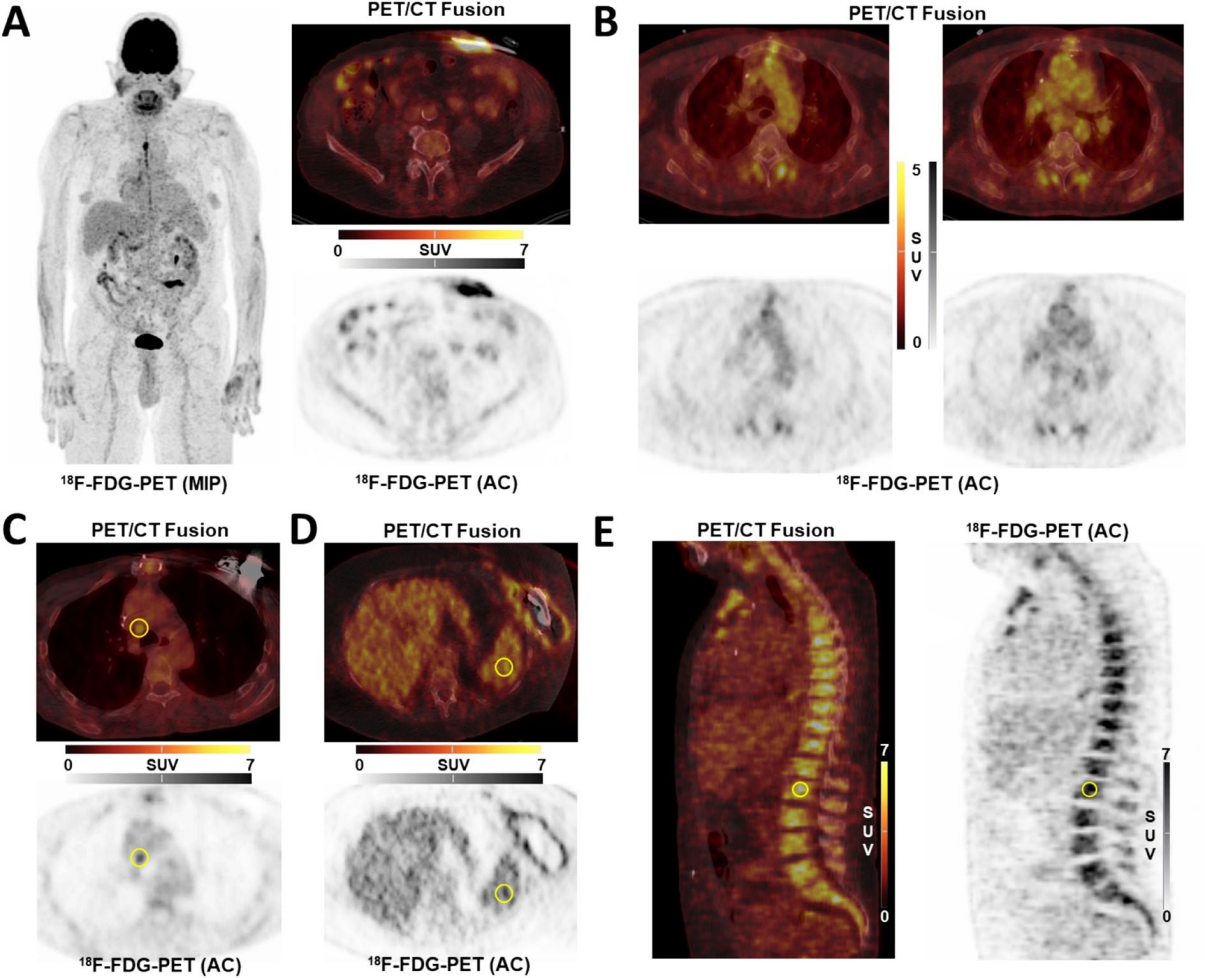
(**A**) Infection of driveline entry site; (**B**) Thoracic vessel signal; (**C**) Mediastinal lymph node signal; (**D**) Spleen signal; (**E**) Bone marrow signal; *AC* attenuation-corrected image; ^18^*F-FDG* ^18^F-fluorodeoxyglucose; *MIP* maximum-intensity projection; *PET/CT* positron emission tomography/computed tomography.

For arterial wall signal, semiquantitative analysis was performed by obtaining the average maximum standardized uptake value (SUV_max_) from 10 separate transaxial slices using circular regions-of interest (ROIs) in each arterial segment and then averaged, yielding a whole-vessel uptake, as previously described^15,23^. Ascending aorta, aortic arch, and descending thoracic aorta were analyzed (Fig. 1B).

For LVADs, the subdivision into the four components (driveline entry point (Fig. 1A), subcutaneous driveline pathway, pump and outflow graft) was made according to the consensus-based standardization of definitions in LVAD infections of the International Society for Heart and Lung Transplantation (ISHLT)^22^. Inflammatory signal of components (SUV_max_) was assessed using the region-of-interest technique as previously described^13^. All scans were screened for sternal and pulmonary co-infection.

For organ interplay, the organ signal in spleen (Fig. 1D), mediastinal lymph nodes (Fig. 1C) and bone marrow (Fig. 1E) was determined^15^. In short, the average of three independent SUV_mean_ measurements in spleen and lumbar vertebra was used for further analyses, as well as SUV_max_ of the mediastinal lymph node with the greatest metabolic activity. PET/CT readers were blinded to clinical outcomes.

### Statistical methods

Data were analyzed using IBM SPSS-Statistics for Windows (v.28.0.0.0 (190); IBM-Deutschland, Munich, Germany). Kolmogorov–Smirnov test was used to test for distribution. Normally distributed continuous variables are given as mean ± SD and non-normally distributed continuous variables as median with interquartile range (IQR). For group comparison of normally distributed variables, the t-test was used. Nonparametric Mann–Whitney U test was used for comparison of groups when normal distribution could not be confirmed. For statistical differences of nominally or ordinally scaled variables, the Chi-Square test was performed. Multiple regression was used to test the influence of several independent variables on a dependent variable. Linear regression was placed for metric dependent variables and binary logistic regression for dichotomous dependent variables. Correlation analysis was accomplished with the Spearman rank correlation test. Kaplan–Meier analysis was used to show predictors of outcome. Hazard ratios (HRs) with 95% CIs were generated. The hazard function with 95% CI adjusted for covariates was plotted to show the mortality and CVE risk during the study period. Receiver operating characteristic (ROC) curve was generated to illustrate the diagnostic value, given as area under the curve (AUC), of the activity of the driveline entry site and subcutaneous driveline pathway and of CRP to predict mortality and stroke. Sub-analysis of longitudinal ^18^F-FDG-PET/CT data were obtained by one-way ANOVA. Statistical significance was defined as a *P* value of < 0.05.

## Results

### Baseline characteristics and topography of LVAD infections

Baseline characteristics of the study population are shown in Table 1. The median interval between LVAD implantation and initial ^18^F-FDG-PET/CT was 700 (IQR: 343–1589) days.

**Table 1:** Baseline characteristics of the entire cohort at time of PET/CT and comparison between patients with and without driveline infection in PET/CT.

Clinical characteristics	All patients n = 118	Driveline infection at baseline PET/CT n = 92	No driveline infection at baseline PET/CT n = 26	*p*
Baseline characteristics				
Age [years] at time of LVAD implantation	54.17 ± 12.197	54 ± 12.156	54.77 ± 12.564	0.783
Sex (male)	101 (85.6%)	78 (84.8%)	23 (88.5%)	0.637
LVAD carrying time prior to PET/CT [days]	700 (343.25–1588.5)	654 (333.75–1593.5)	847 (347–1649)	0.773
Etiology of heart disease				0.196
Dilated CM	66 (55.9%)	55 (59.8%)	11 (42.3%)	0.113
Ischemic CM	41 (34.7%)	30 (32.6%)	11 (42.3%)	0.359
Other	11 (9.4%)	7 (7.6%)	4 (15.4%)	0.229
Type of LVAD				0.662
HeartMate 3	41 (34.7%)	33 (35.9%)	8 (30.8%)	0.630
HeartMate II	16 (13.6%)	13 (14.1%)	3 (11.5%)	0.733
HeartWare	56 (47.5%)	41 (44.6%)	15 (57.7%)	0.237
Other	5 (4.2%)	5 (5.4%)	0 (0%)	0.224
Implant technique (minimally invasive)	59 (50%)	42 (46%)	16 (61.5%)	0.112
Arterial hypertension	83 (70.3%)	60 (65.2%)	23 (88.5%)	0.022*
MAP at time of PET/CT in mmHg	79.15 ± 11.915	79.05 ± 12.646	79.5 ± 9.187	0.855
Diabetes mellitus	43 (36.4%)	36 (39.1%)	7 (26.9%)	0.253
Hyperlipidaemia	69 (58.6%)	53 (57.6%)	16 (64%)	0.647
History of smoking	76 (64.4%)	58 (63%)	18 (69.2%)	0.626
Pack years	25 (4–40)	24 (4–36.25)	30 (4.5–45)	0.565
Statins	40 (33.9%)	30 (32.6%)	10 (38.5%)	0.578
Driveline entry site with clinical apparent infection	65 (55.1%)	62 (67.4%)	3 (12%)	< 0.001*
CRP at time of PET/CT in mg/l	12.3 (5.35–28.425)	11.4 (5.25–24.775)	15.95 (5.18–51.275)	0.220
WBC at time of PET/CT in 10^3^/µl	7.55 (6–9.1)	7.6 (6.025–9.4)	7.3 (5.98–8.45)	0.445
Outcome				
Device exchange	40 (33.9%)	32 (34.8%)	8 (30.8%)	0.703
Death	47 (39.8%)	35 (38%)	12 (46.2%)	0.456
Stroke (%)	22 (18.6%)	19 (20.7%)	3 (11.5%)	0.292
Ischemic	15 (12.7%)	14 (16.1%)	1 (3.8%)	0.130
Haemorrhagic	8 (6.8%)	16 (18.4%)	2 (7.7%)	0.237
Postoperative	4 (3.4%)	4 (4.3%)	0 (0%)	0.121
Heart transplantation	23 (19.5%)	20 (21.7%)	3 (11.5%)	0.246
Infection of pump pocket	38 (32.2%)	30 (32.6%)	8 (30.8%)	0.859
Infection of outflow graft	43 (36.4%)	37 (40.2%)	6 (23.1%)	0.109

Results are displayed as frequencies (percentages), metric data as mean ± SD if normally distributed or median with interquartile range (25–75th percentile) if not normally distributed. Significant values are in bold.

*IQR* Interquartile range, *LVAD* left ventricular assist device, *CM* cardiomyopathy, *CRP* C-reactive protein, *PET/CT* positron emission tomography/computed tomography, *MAP* mean arterial pressure, *WBC* white blood cell count.

*p* < 0.05 is considered statistically significant. In four patients CRP and CBC were missing.

**Figure 2:**
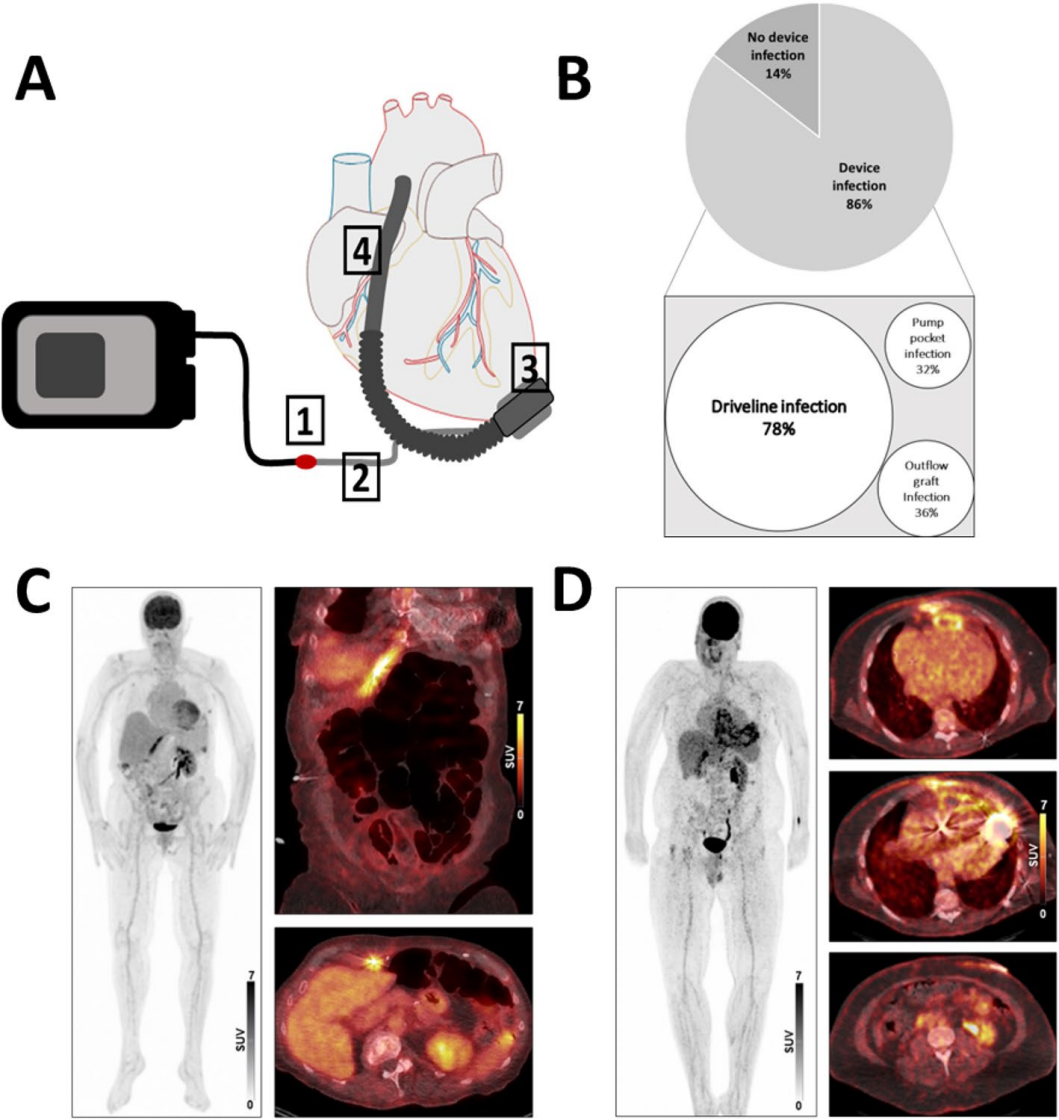
(**A**) Schematic illustration of the LVAD system, subdivided in 4 components: (1) driveline entry site, (2) subcutaneous driveline pathway, (3) pump pocket, (4) outflow graft; (**B**) Infection topography; (**C**) Infection of subcutaneous driveline pathway; (**D**) Infection of subcutaneous driveline pathway and thoracic components.

DLIs were detected in 92/118 (78%) patients by ^18^F-FDG-PET/CT. Irrespective of DLI, an exclusive infection of the internal LVAD-components was found in 10/118 (8%). It is noteworthy that with 65/118 (55%), only half of the entire cohort presented with clinically apparent DLI. As an important finding, DLI could be identified by PET/CT in 30/53 (57%) patients who had a clinically inapparent driveline entry sites. Besides increased metabolic activity at the driveline entry site or the subcutaneous driveline pathway, in 43/118 (36%) infection of the outflow graft and in 38/118 (32%) the pump pocket was found (Fig. 2B). At the time of PET/CT, 75/118 (64%) patients had a positive microbiological smear at the driveline entry site. The pathogens most frequently detected were Staphylococcus aureus in 19/75 (25%), Staphylococcus epidermidis and Pseudomonas aeruginosa in 13/75 (17%) each, aerobic gram-positive bacilli in 8/75 (11%), enterobacteria in 7/75 (9%), Corynebacterium amycolatum, Escherichia coli and Serratia marcescens in 5/75 (5%) each. Bloodstream infections were detected in 13/118 (11%) patients, 3 of whom had VAD-related bloodstream infections. Compared to patients with negative blood culture results, those with positive results showed increased levels of CRP (*p* < 0.001) and WBC (*p* = 0.02). At the time of PET/CT, 83/118 (70%) patients were already covered by antibiotic therapy due to a LVAD-specific infection or an unknown infection focus with an indication for empiric antibiotic therapy. No patient was in the intensive care unit at the time of infection diagnosis. A comparison of PET/CT parameters between the patients with and without driveline infection in PET/CT is shown in Table 2. CRP levels correlated with metabolic tissue activity close to internal components such as the pump pocket (r = 0.27, *p* = 0.003) and the outflow graft (r = 0.19, *p* = 0.047), but less with the subcutaneous driveline (r = 0.17, *p* = 0.073) and not with the driveline entry site (r = -0.08, *p* = 0.40). Also, CRP levels correlated with imaging-derived systemic inflammatory activity (spleen signal (r = 0.22, *p* = 0.022), bone marrow signal (r = 0.22, *p* = 0.021), and mediastinal lymph node signal (r = 0.27, *p* < 0.001)). Of note, only 21/89 (24%) patients with DLI on PET/CT showed CRP levels below the upper reference limit (5 mg/L), but 82/88 (93%) patients had a normal WBC (< 10,5 × 10^9^/L). Fever (> 38 °C) was found in 1/82 (1%) of the patients at the time of the PET/CT-diagnosed infection. Pulmonary infiltrates as a competing focus of infection and cause of an increase in inflammation parameters were detected in 9/118 (8%) patients in PET/CT. This group showed a significantly higher CRP (10.4 mg/L (4.6–26) vs. 32.1 mg/L (18.3 – 99.2), *p* = 0.004).

**Table 2:** PET/CT parameters of the entire cohort and comparison between patients with and without driveline infection in PET/CT.

Clinical characteristics	All patients n = 118	Driveline infection at baseline PET/CT n = 92	No driveline infection at baseline PET/CT n = 26	*p*
Period between initial PET/CT and death in days	366 (125 – 743)	366 (147 – 752)	349 (101 – 653)	0.354
Average SUV_max_ thoracic vessels	2.9286 ± 0.4891	2.9544 ± 0.4875	2.7969 ± 0.5196	0.172
SUV_max_ subcutaneous driveline pathway	8.52 (5.62 – 12.92)	10.0167 ± 4.5796	6.5744 ± 4.2371	< 0.001*
SUV_max_ driveline entry point	4.71 (2.64 – 8.415)	6.045 (3.575 – 9.698)	2.33 (1.9 – 3.095)	< 0.001*
SUV_max_ outflow graft	6.245 (4.93 – 9.06)	6.43 (4.735 – 9.238)	5.91 (5.258 – 7.803)	0.548
SUV_max_ pump pocket	7.455 (5.245 – 10.37)	7.42 (5.17 – 10.64)	8 (6.155 – 9.78)	0.907
SUV_max_ sternum	6.86 (4.99 – 9.31)	7.23 (5.22 – 9.64)	5.92 (4.17 – 7.58)	0.041*

Results are displayed as frequencies (percentages), metric data as mean ± SD if normally distributed or median with interquartile range (25–75th percentile) if not normally distributed. *p* < 0.05 is considered statistically significant; *PET/CT * positron emission tomography/computed tomography, *SUV * standardized uptake value, *IQR* Interquartile range. Significant values are in bold.

WBC count did not correlate with any imaging-derived inflammatory parameters.

Sternal co-infection was associated with pump pocket infection (*p* < 0.001, OR = 7.25, 95% CI  2.61–20.11), outflow graft infection (*p* = 0.005, OR = 3.8, 95% CI  1.44–10.01), number of infected LVAD components (*p* = 0.002) and PET/CT signal of mediastinal lymph nodes (*p* = 0.031).

### Organ interplay

**Figure 3:**
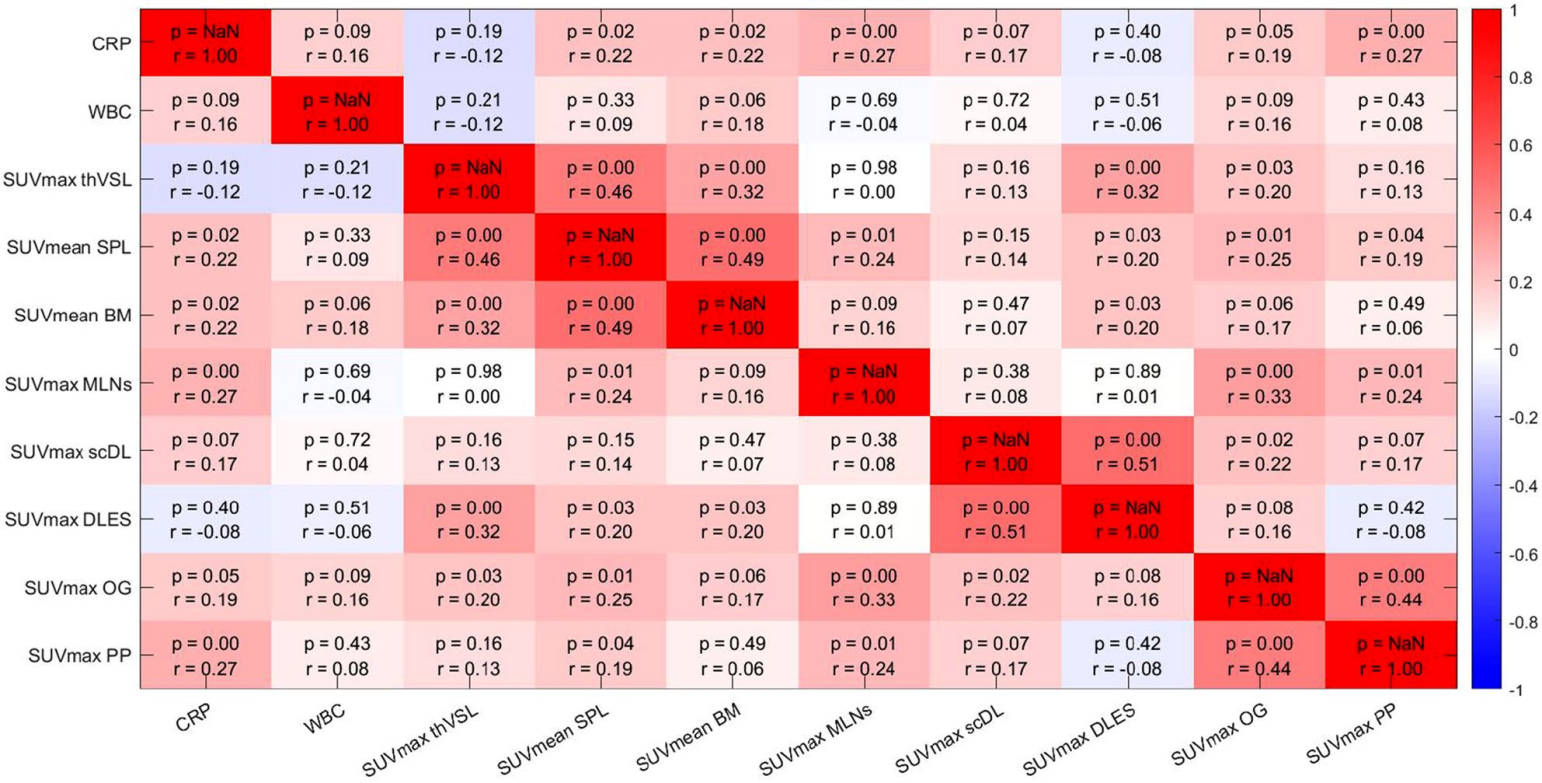
Heatmap: Spearman correlation of the parameters of the first PET/CT, n = 118, in four patients CRP and CBC were missing; blue: r = -1, red: r = 1; *CRP* C-reactive protein, *WBC* white blood cell count, *SUV* standardized uptake value, *thVSL* thoracic vessels, *SPL* spleen, *BM* bone marrow, *MLNs* mediastinal lymph nodes, *scDL* subcutaneous driveline pathway, *DLES* driveline entry site, *OG* outflow graft, *PP* pump pocket.

Correlation analysis of PET signal with device components, activity of aortic vessel walls and lymphoid and hematopoietic organs is shown in Fig. 3. Metabolic activity at the driveline entry site correlated with a) the aortic vessel signal (r = 0.32, *p* < 0.001) and b) both lymphoid organs, namely spleen signal (r = 0.20, *p* = 0.030), and bone marrow signal (r = 0.20, *p* = 0.030), highlighting systemic interactions. Metabolic activity of the aortic vessel walls itself correlated with spleen signal (r = 0.46, *p* < 0.001) and bone marrow signal (r = 0.32, *p* < 0.001).

Multivariable analysis including metabolic activity at the driveline entry site, aortic vessel wall activity, spleen signal and bone marrow signal revealed an independent association between aortic vessel wall activity and metabolic activity at the driveline entry site (β = 0.04, 95% CI 0.01–0.06, *p* = 0.001) and spleen signal (β = 0.43, 95% CI 0.18–0.68, *p* < 0.001).

A longitudinal sub-analysis of repeated PET/CTs was performed to investigate potential changes in metabolic activity in the vessel wall, revealing stability over time (ANOVA, *p* = 0.27).

### Cerebrovascular outcome events

Within the observation period, 22/118 (19%) patients suffered a CVE, of which 15/22 (68%) had an ischemic stroke and 8/22 (36%) a haemorrhagic stroke. No patient suffered a transient ischemic attack (TIA) during the follow up period. One patient suffered both, an ischemic and haemorrhagic stroke. Seven patients had 2 or more CVE and 11/22 (50%) died within a median of 263 days after the CVE (Table 3).

**Table 3:** Stroke characteristics of patients suffering CVE under LVAD therapy after the initial PET/CT.

Characteristics	Patients with CVE under LVAD therapy n = 22 (18.6%)
Number of CVE per patient 1 2 3	13 (65%) 5 (25%) 2 (10%)
Heamorrhagic stroke Ischemic stroke Ischemic and heamorrhagic stroke Transient ischemic attack	7 (31.8%) 14 (63.6%) 1 (4.5%) 0 (0%)
Postoperative stroke	4 (18.2%)
Anterior circulation Posterior circulation Both circulations	20 (91.7%) 2 (8.3%) 0 (0%)
Stroke etiology based on TOAST criteria (%): Large-artery atherosclerosis Cardioembolism Small-vessel occlusion Other determined etiology Undetermined etiology	0 (0%) 20 (91.7%) 0 (0%) 0 (0%) 0 (0%)
Deceased (%)	11 (50%)
Time interval between (last) stroke and death in days	263 (22 – 755)

Results are displayed as frequencies (percentages), metric data as mean ± SD if normally distributed or median with interquartile range (25th-75th percentile) if not normally distributed.

*CVE* cerebrovascular event, *TOAST* Trial of Org 10,172 in Acute Stroke Treatment, *IQR* Interquartile range.

**Figure 4:**
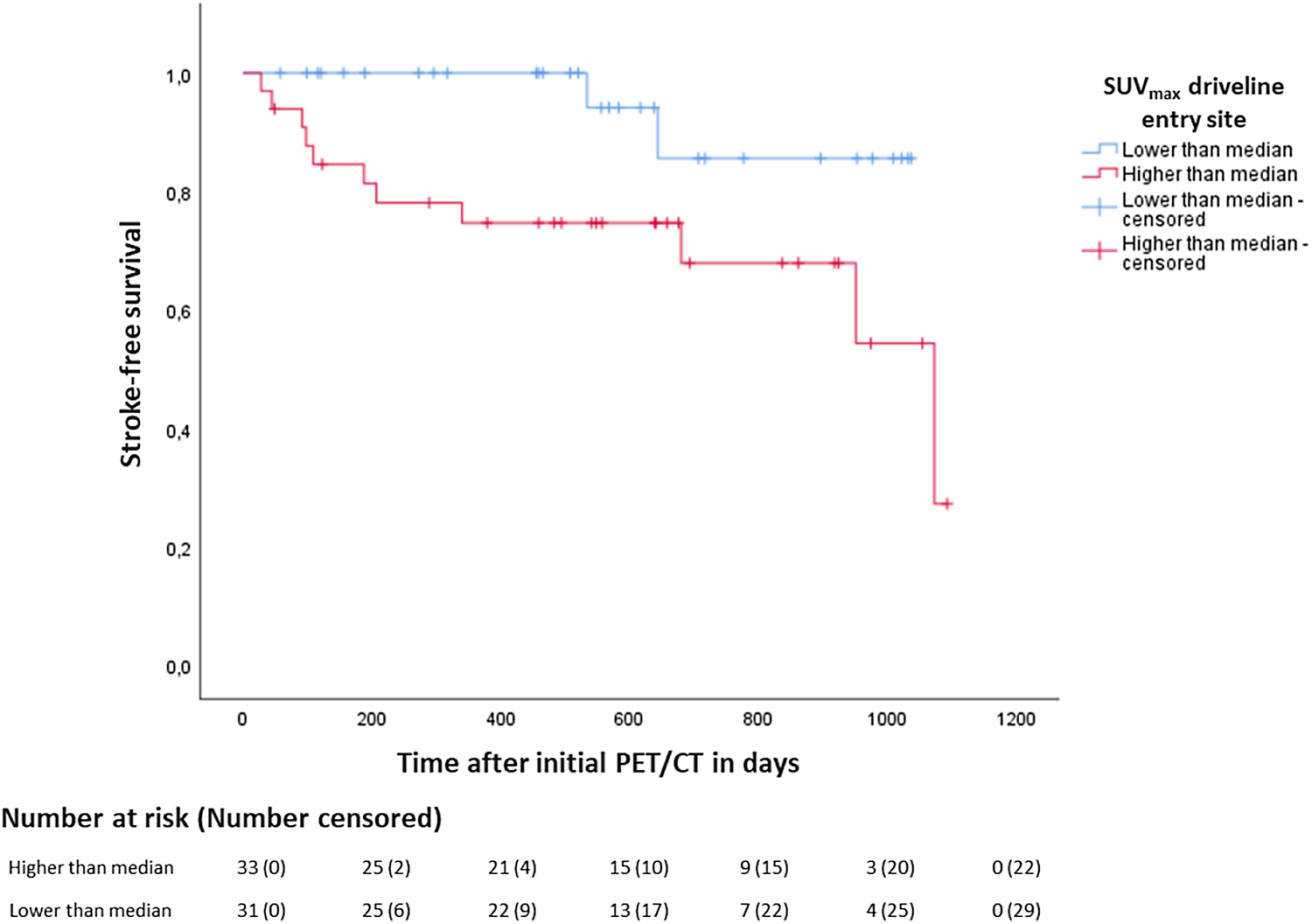
Kaplan Meier analysis on ischemic stroke-free survival; differences in the incidence of ischemic stroke between patients with SUV_max_ of the driveline entry site above versus below the median. The analysis revealed a significantly differing hazard for ischemic stroke (χ^2^(1) = 5.21, *p* = 0.023; HR = 10.21, 95% CI  1.64–63.52, *p* = 0.013).

Characteristics of patients with ischemic stroke (n = 15) compared with those without ischemic stroke (n = 96) after excluding patients with haemorrhagic stroke (n = 7) are shown in Table 4. The group with ischemic stroke after PET/CT demonstrated significantly higher metabolic activity at the driveline entry site (*p* = 0.04), whereas the activity of other device components or CRP and WBC did not differ. In multivariable analysis adjusting for type of anticoagulant (VKA, heparin, others) and device exchange, higher metabolic activity at the driveline entry site was not significantly associated with ischemic stroke (OR = 1.16, 95% CI 1–1.33, *p* = 0.05). The type of anticoagulation (OR of heparin or others vs. VKA = 3.22, 95% CI 1.31–7.88, *p* = 0.01) proved to be an independent predictor for ischemic stroke. The type of anticoagulation in LVAD patients is often modified in the course of an inpatient stay and can therefore serve as an indicator for hospitalization with severe comorbidity, e.g., bleeding complications. Consequently, not only anticoagulation itself is a relevant competing cause of stroke, but also an indication of other contributing risk factors. The severity of infection as measured by fever (*p* = 0.505), number of infected LVAD components on PET/CT (*p* = 0.232) and laboratory parameters showed no association with the occurrence of CVEs. At the time of stroke, 14/22 (64%) patients were hospitalized due to following concomitant conditions: acute pump stop (n = 1), driveline infection (n = 6), gastrointestinal hemorrhage (n = 1), heart transplant (n = 2), device thrombosis (n = 3) with indication for device exchange and domestic fall (n = 1). In this context, VKA was paused in some patients, and anticoagulation was switched to heparin (n = 6) or Argatroban (n = 2). At the time of ischemic stroke, 8/15 (53%) patients were anticoagulated with vitamin K antagonists, four of whom were within, one was below and three were above the intended target International Normalized Ratio (INR) range. 7/15 (47%) patients were on heparin therapy under activated Partial Thromboplastin Time (aPTT) control, of which three were below and one was above the target aPTT range. At the time of haemorrhagic stroke, 6/7 (86%) patients were receiving anticoagulation with vitamin K antagonists, with one patient within and five patients above the intended target INR range. 1/7 (14%) patient was receiving heparin therapy and was within the target aPTT range. Ischemic stroke occurred more frequently in patients with Heparin/Argatroban compared to those who remained on VKA (*p* < 0.001). In a log-rank test the incidence of ischemic stroke differed between patients with SUV_max_ of the driveline entry site above the median compared with those under the median (Fig. 4; χ^2^(1) = 5.21, *p* = 0.023). The hazard of ischemic stroke, adjusted for type of anticoagulation, hyperlipidemia and diabetes mellitus, was about 10 times higher in the group with an increased uptake of the driveline entry site (HR = 10.21, 95% CI 1.64–63.52, *p* = 0.013). Receiver operating characteristics (ROC) analysis demonstrated the diagnostic ability of the activity of the driveline entry site to predict ischemic stroke after initial PET/CT (Fig. 1 in supplement; AUC = 0.67, *p* = 0.04).

**Table 4:** Comparison of patients with and without ischemic stroke after PET/CT, excluding haemorrhagic stroke.

Clinical characteristics	Patients with ischemic stroke n = 15	Patients without ischemic stroke n = 96	*p*
Age at time of LVAD implantation in years	56.07 ± 9.61	54 ± 12.36	0.538
Sex (male)	13 (86.7%)	83 (86.5%)	0.982
Time on LVAD prior to PET/CT in days	729 (419 – 1421)	600.5 (315.5 – 1578.5)	0.540
Etiology of heart disease			0.276
Dilated CM	7 (46.7%)	53 (55.2%)	0.537
Ischemic CM	7 (46.7%)	33 (34.4%)	0.356
Other	1 (6.7%)	10 (10.4%)	0.651
Type of LVAD			0.951
HeartMate 3	6 (40%)	34 (35.4%)	0.731
HeartMate II	2 (13.3%)	13 (13.5%)	0.982
HeartWare	7 (46.7%)	45 (46.9%)	0.988
Other	0 (0%)	4 (4.2%)	0.421
Arterial hypertension	11 (73.3%)	69 (71.9%)	0.907
Diabetes mellitus	3 (20%)	35 (36.5%)	0.212
Hyperlipidaemia	10 (66.7%)	55 (57.3%)	0.493
History of smoking	10 (66.7%)	60 (62.5%)	0.927
Pack years	25 (7.5 – 62.5)	30 (5 – 40)	0.855
Myocardial infarction	8 (53.5%)	33 (34.4%)	0.157
Statins	7 (46.7%)	31 (32.3%)	0.275
Device change	8 (53.5%)	28 (29.2%)	0.063
Death	8 (53.5%)	36 (37.5%)	0.244
CRP at time of PET/CT in mg/l	6.3 (3.3 – 20.6)	13.55 (5.25 – 34.4)	0.204
WBC at time of PET/CT in 10^3^/µl	7.6 (6.6 – 8.3)	7.45 (6 – 9.05)	0.893
SUV_max_ subcutaneous driveline pathway	10.47 (7.15 – 13.55)	7.72 (5.07 – 12.85)	0.086
SUV_max_ driveline entry point	6.36 (5.23 – 11.74)	4.44 (2.56 – 8.26)	0.04

Results are displayed as frequencies (percentages), metric data as mean ± SD if normally distributed or median with interquartile range (25th-75th percentile) if not normally distributed. *p* < 0.05 is considered statistically significant. In four patients CRP and CBC were missing.

*LVAD* left ventricular assist device, *PET/CT* positron emission tomography/computed tomography, *CM* cardiomyopathy, *CRP* C-reactive protein.

A log-rank test was used to determine the incidence of CVE for the comparison of SUV_max_ of the subcutaneous driveline pathway above versus below the median. The incidence of stroke differed significantly between the two groups (Fig. 2 in supplement; χ^2^(1) = 6.76, *p* = 0.009). The hazard of suffering a CVE was 3.5 times greater in the group with an increased uptake of the subcutaneous driveline pathway (HR = 3.53, 95% CI 1.28–9.72, *p* = 0.015).

### Mortality

During the observation period, 47/118 (40%) patients died within 1715 (± 1050) days after implantation. Causes of death were sepsis (50%), right heart failure (17%), hemorrhage (6%), pump thrombosis (6%) and brain herniation after intracerebral hemorrhage (4%) or middle cerebral artery infarction (2%). Cause of death was unknown for 9%.

Deceased patients showed more often hyperlipidemia (*p* = 0.008), increased CRP values (*p* = 0.001) and history of device exchange (*p* = 0.016).

In addition, metabolic activity of the subcutaneous driveline pathway (*p* = 0.005) and thoracic lymph node signal were significantly higher in deceased patients (*p* = 0.017). Binary logistic regression analysis including metabolic activity of the subcutaneous driveline pathway, hyperlipidemia, mediastinal lymph node signal and device exchange revealed increased mortality in patients with hyperlipidemia (OR = 1.90, 95% CI 1.29–2.79, *p* = 0.001) and higher metabolic activity of the subcutaneous driveline pathway (OR = 1.13, 95% CI 1.02–1.24, *p* = 0.016). No association was found between mortality and severity of infection as measured by fever (*p* = 0.232), number of infected LVAD components in PET/CT (*p* = 0.268) and laboratory parameters.

**Figure 5:**
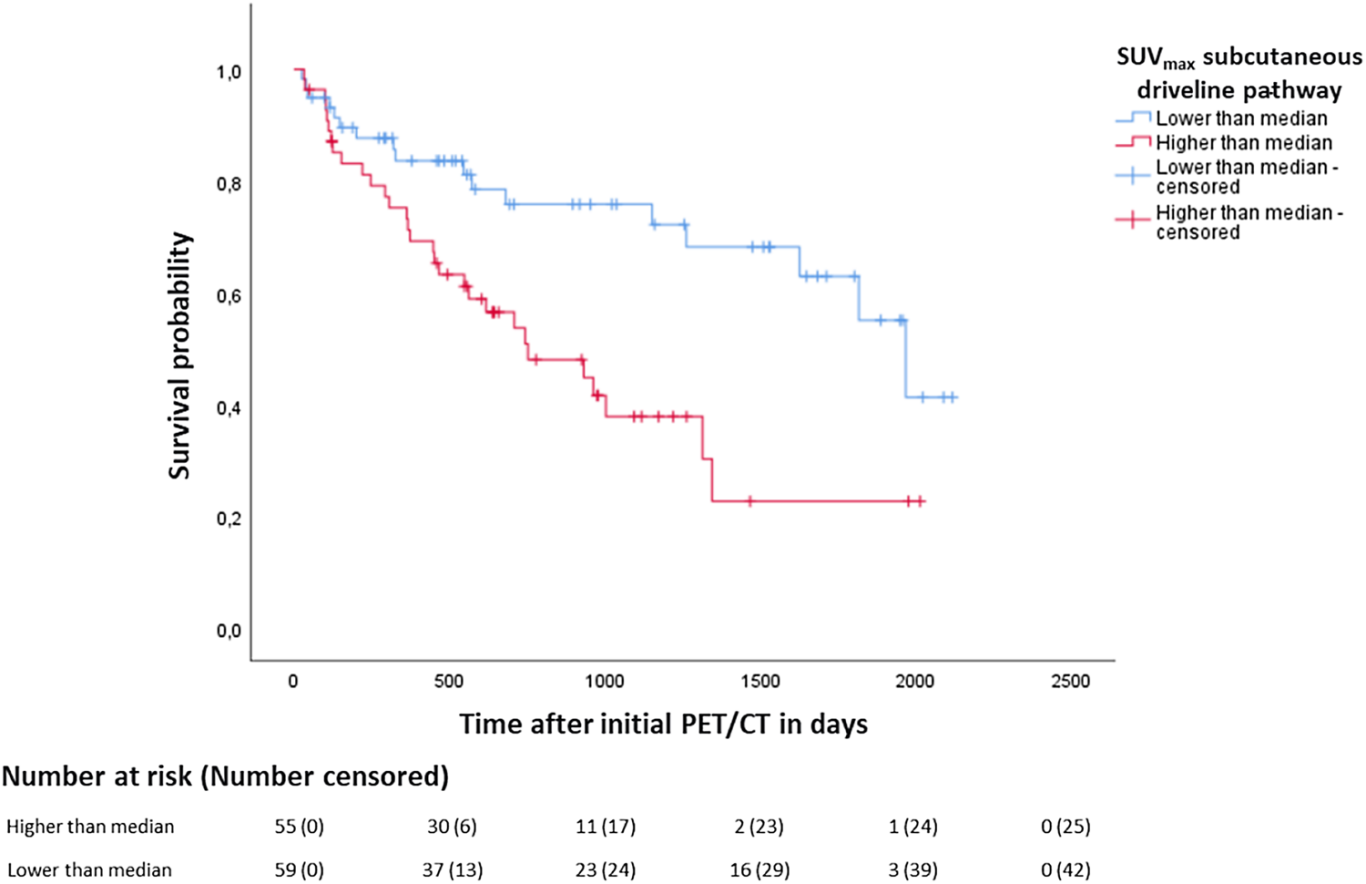
Kaplan Meier survival analysis: Mortality; differences in survival between patients with SUV_max_ of the subcutaneous driveline pathway above versus below the median. The analysis revealed a significantly differing distribution of survival (χ^2^(1) = 9.18, *p* = 0.002; HR = 1.13, 95%CI  1.05–1.21, *p* < 0.001).

A log-rank test revealed a significant difference in survival of patients with metabolic activity of the subcutaneous driveline pathway above the median compared with those below the median (Fig. 5; χ^2^(1) = 9.18, *p* = 0.002). Patients with increased subcutaneous driveline metabolic activity showed a 13% increase in the risk of mortality in Cox regression analysis after adjustment for age, diabetes mellitus and hyperlipidaemia (HR = 1.13, 95% CI  1.05–1.21, *p* < 0.001).

We performed a ROC analysis for prediction of mortality (Fig. 2, 3 in supplement). The analysis showed that both, CRP in the morning of the first PET/CT (Fig. 3; AUC = 0.68, *p* = 0.001) and metabolic activity of subcutaneous driveline (Fig. 4 in supplement; AUC = 0.65, *p* = 0.005), can help to predict mortality.

## Discussion

With this retrospective exploratory analysis of 118 LVAD patients, we intended to investigate the impact of device infections on systemic and local inflammatory responses in LVAD patients using ^18^F-FDG-PET/CT. The main findings of our study were (i) systemic inflammation response with activation of hematopoietic and lymphoid organs, potentially triggered and maintained by device-associated infections, (ii) a strong association between infection of the driveline and local vascular inflammatory responses, (iii) and driveline infections as predictive marker for mortality and stroke. As part of this inflammatory interplay, an association between DLI and enhanced arterial metabolic activity could be established for the first time.

It should be noted that DLIs in our study cohort were not always clinically detectable with an inconspicuous driveline entry site or laboratory tests of WBC and CRP. In contrast, CRP levels were elevated in patients with pulmonary infiltrates, hence an increase in CRP levels might indicate a non-VAD infection, e.g., pneumonia. However, the discrepancy between clinical apparent infections and metabolic activity could be due to local irritation of the driveline caused by mechanical stress. Apart from sterile infections, the discrepancy between clinical and PET/CT diagnosed DLI might be explained by subcutaneous and thus hidden material, but also by pre-existing antibiotic therapy. Furthermore, the LVAD material is also predestined for local, encapsulated infections. For this reason, the high sensitivity (> 90%) and specificity (> 90%) of ^18^F-FDG PET/CT for detecting LVAD-specific infections is of great importance^24^. In a previous study, infection was uncovered by PET/CT in 55% of 35 LVAD patients, despite a clinically unremarkable driveline entry site^25^. These findings are consistent with our data, in which infection was detected in 57% of 53 patients with clinically inapparent driveline entries^13^. Of note, PET/CT detected DLI in 30 patients with clinically inapparent driveline and found infection of the internal LVAD components in 8% without DLI. Laboratory inflammation parameters were not significantly elevated in patients with DLI, thus PET/CT examination offers the only minimally invasive opportunity to immediately detect infection of the internal LVAD-components as well as inapparent DLI. By diagnosing infection by PET/CT, appropriate therapy can be initiated instantly and the enumerated complications associated with LVAD-specific infections can be avoided.

We also explored a systemic inflammatory response to local infection using imaging-based parameters such as spleen, bone marrow and thoracic lymph node signal^13,15^. We observed increased metabolic activity in spleen and bone marrow – the major hematopoietic and lymphoid organs. This increased metabolic activity is associated with the release of inflammatory cells and progenitor cells but is also associated with atherosclerosis^16,26^. Indeed, the splenic metabolic activity itself has also been shown to be a predictor of cardiovascular events, independent of pre-existing risk factors^26^. The cardiosplenic axis as well as the spleen-atherosclerotic plaque axis are responsible for the link between spleen activity and cardiovascular events. The spleen is a key contributor to the release of proinflammatory monocytes and leukocytes. These spleen-derived inflammatory cells induce vascular inflammation and were also found in atherosclerotic plaques^26^.

Another major finding of our study is the increased metabolic activity of the aortic wall in patients with DLIs. In a previous study including oncologic patients, increased ^18^F-FDG uptake in the major arteries emerged as the strongest predictor of a subsequent vascular event, and increased wall signal in this cohort indicated that local inflammation is driving a systemic inflammatory response that also modulates inflammatory cell activity in the aortic wall^27^. This might add to the knowledge on inflammation-modulated vascular changes and remodelling under mechanical circulatory support. Specific modifications of the vessel walls and fibrotic changes have already been observed histologically under LVAD-support^28,29^. In the exploratory longitudinal sub-study, aortic wall signal remained stable, albeit in a small fraction of patients and with non-standardized time points for repeated PET.

Irrespective of infection-driven systemic inflammation, various reasons for a permanently increased systemic inflammatory baseline level must be considered in LVAD patients, such as reduced ejection fraction^30,31^, myocardial damage-induced cytokine release^32^, mitochondrial damage caused by oxidative stress^33,34^, artificial surfaces^32^ and shear stress^32^. Independent of the known chronic predisposing factors, vascular metabolic activity in PET/CT was associated with concurrent DLI in our study, suggesting a systemic trigger by infection reflected by vessel wall inflammation.

Furthermore, chronic bacterial infection is known to be associated with the development, progression, and destabilisation of atherosclerosis, being a common comorbidity in LVAD patients^35,36^. Not only direct infection of the vessel wall, but also the influence of a nearby local infection, such as periodontitis or urinary tract infection, has been described^35,36^. The phenomenon of triggering an acute arterial inflammatory reaction as a by-product of an extravascular infectious focus is called the “echo” effect^36^. In our study, the local infection of the driveline might also trigger an inflammatory response in the thoracic aorta.

In our preselected cohort with a large number of DLIs, ischemic and haemorrhagic strokes occurred with an incidence of 19% during a relatively long observation period. This is quite reasonable, whereas in large surveys of LVAD patients, the lifetime prevalence of stroke ranges from 7 to 13% in LVAD patients^37,38^.

Previous studies have reported an increased risk of both, ischaemic and haemorrhagic stroke, associated with device-related infections and DLIs^6,39,40^, which may indicate an influence of inflammatory activity represented by the metabolic activity of the driveline on stroke risk. In our study, we only found a trend without significant evidence between increased metabolic activity of the driveline and the occurrence of CVE. The precision of estimates was low for CVE analyses most likely due to the small study population. Therefore, the findings must be interpreted with caution. Mechanisms being discussed include endothelial activation^41^, immunothrombosis^42,43^ and hypercoagulability^44^. These multifactorial influencing factors explain why no direct correlation between vascular activation and CVE could be found in our study. While the pathogenesis of infection-related ischemic stroke is well understood and is mainly caused by the formation of microemboli through activation of leukocytes, complement system and acute phase proteins, the pathogenesis of haemorrhagic stroke is not yet fully understood. Inflammation and infection of the vessels may lead to instability of the cerebral microvascular system and trigger bleeding or formation of septic aneurysms^40^.

However, in some cases, the occurrence of stroke is significantly distant in time from performed PET/CTs with evidence of DLI, so that a direct causal relationship seems unlikely.

It is striking that 64% of patients were hospitalised at the time of stroke due to bleeding, pump thrombosis, hypertension and atrial fibrillation presenting pre-described risk factors for ischemic strokes^6,39^. Consecutive adjustments in anticoagulation and concomitant conditions additionally pose according competing risks for stroke^45^.

For instance, as evidenced by data from the Intermacs Annual Report, infections play a predominant role in mortality in LVAD patients^3^. Systemic inflammation is known to constitute a contributing risk factor for increased mortality^46^. In our cohort, a preceding systemic inflammation was uncovered by imaging data, and consequently sepsis was the leading cause of death, accounting for about 50% of deaths. However, a shift in the distribution of outcomes due to the preselected collective must be considered, which impedes comparisons with previous studies. Besides, this cohort showed a significant association between mortality and hyperlipidaemia. Hyperlipidaemia has already been described as an influencing factor for poor outcome after heart transplantation as well as for disease progression^47^ and for adverse events in other cardiovascular diseases, mainly through immune activity, e.g., by affection of haematopoietic stem cells and leukocytes^48^. Lipid lowering with statins reduces these immunomodulatory effects^47^.

Our analyses showed that metabolic activity of the driveline was accompanied with an increased risk of death, which is in accordance with previous studies^49^. Regardless of our findings, it remains to be debated whether LVAD-specific infections are the sole driving and leading cause of stroke and death or whether they are more likely a player in a concert of multiple risk factors that undermine the balanced status in these vulnerable patients. In view of the latest clinical trials^50^ as well as the annual INTERMACS registries^51^ mortality, LVAD thrombosis and strokes decreased in patients with HeartMate3. We could not see any statistically relevant differences when investigating the type of device for several AEs, which might be due to the small sample size.

Some limitations should be acknowledged. First, the retrospective nature of our study comes along with inherent limitations. Second, we investigated infection-driven changes in the aortic wall and organs in the setting of DLI/LVAD infection, but not vascular changes under circulatory support without infection nor their changes in a control group of patients with heart failure without LVAD. However, due to a strikingly frequent and chronic course of infections in LVAD patients and their reciprocal interplay with adverse events, our patient group might be representative for a large portion of LVAD patients with severely affected outcomes. Besides, in this study, the median time between the last PET/CT scan and death as most important outcome event was 120 days, ranging from 3 to 1966 days, advocating a cautious interpretation of the link between PET/CT findings and outcome.

## Conclusion

^18^F-FDG-PET/CT allows valuable insights into the inflammatory interplay between large arterial walls, lymphoid and hematopoietic organs and device components in LVAD patients with device-specific infection. Increased FDG signal at the driveline was associated with the occurrence of CVE and mortality, pointing to the urgent need of adequate infection and inflammation control. A clinical assessment appears not sufficient. We recommend the early performance of a PET/CT scan.

### Supplementary Information

Supplementary Information.

## Data Availability

Data that support the findings of this study are available from the corresponding author upon reasonable request.

## Abbreviations

aPTT: Activated partial thromboplastin time
CBC: Complete blood count
CM: Cardiomyopathy
CRP: C-reactive protein
CVE: Cerebrovascular events
DLI: Driveline infection
eGFR: Estimated glomerular filtration rate
^18^F-FDG-PET/CT: ^18^F-fluorodeoxyglucose (FDG)-positron emission tomography/computed tomography
HR: Hazard ratio
INR: International normalized ratio
IQR: Interquartile range
LVAD: Left ventricular assist device
MAP: Mean arterial pressure
OR: Odds ratio
ROC: Receiver operating characteristics
SUV: Standardized uptake value
SD: Standard deviation
TOAST: Trial of Org 10172 in Acute Stroke Treatment
VKA: Vitamin K antagonist
WBC: White blood cell
