# Supplementary material for: Interplay between driveline infection, vessel wall inflammation, cerebrovascular events and mortality in patients with left ventricular assist device
Source: Sci Rep. 2023 Oct 29;13:18552. doi: 10.1038/s41598-023-45110-6 (PMC10613624; doi:10.1038/s41598-023-45110-6)

**Supplement**

**Table of content**

[**Material and methods** 1](#_Toc126783478)

[Materials and methods: Study group and clinical data 1](#_Toc126783479)

[**Results** 1](#_Toc126783480)

[Results: Organ Interplay 1](#_Toc126783481)

[Results: Outcome events: 2](#_Toc126783482)

[**Supplement Figure** 2](#_Toc126783483)

[Supplement Figure 1 2](#_Toc126783484)

[Supplement Figure 2 3](#_Toc126783485)

[Supplement Figure 3 3](#_Toc126783486)

[Supplement Figure 4 4](#_Toc126783487)

**Material and methods**

*Materials and methods: Study group and clinical data*

In four patients CRP and CBC were missing, in three of them eGFR values were not available.

**Results**

*Results: Organ Interplay*

Both, the metabolic activity at the pump pocket correlated with activity of spleen (r=0.19, p=0.044) and mediastinal lymph nodes (SUV_max_, r=0.24, p=0.008) and metabolic activity at the outflow graft also correlated with those of spleen (r=0.25, p=0.008) and mediastinal lymph nodes (SUV_max_, r=0.33, p<0.001).

**Supplement Figure**

Supplement Figure 1: ROC curve ischemic stroke: shows the diagnostic ability of the activity of the driveline entry site for predicting ischemic stroke; SUV_peak_ driveline entry site: AUC 0.7 (0.57-0.83), p=0.013; SUV_max_ driveline entry site: AUC 0.67 (0.52-0.81), p=0.04; SUV_mean_ driveline entry site: AUC 0.67 (0.52-0.81), p=0.041


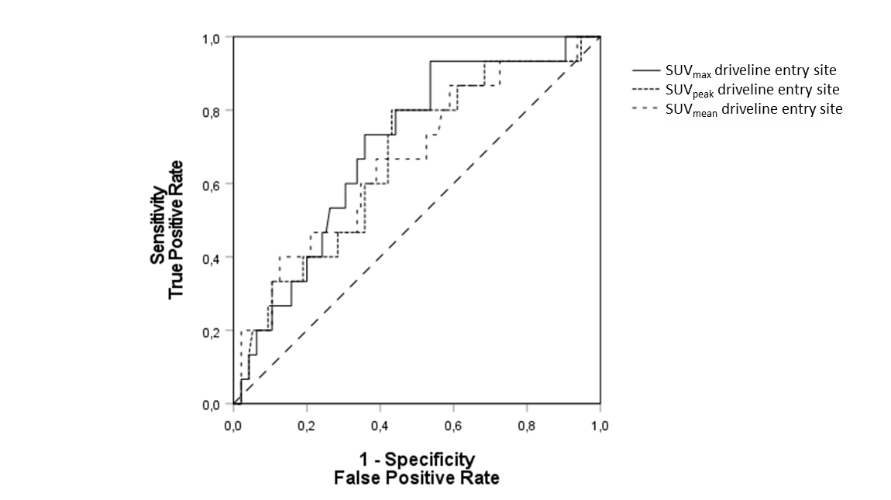


Supplement Figure 2: Kaplan Meier CVE free survival; differences in the incidence of CVE between patients with SUV_max_ of the subcutaneous driveline pathway above vs below the median. The analysis revealed a significantly differing hazard for CVE (χ²(1)=6.76, p=0.009); HR=3.53, 95%CI=1.28-9.72, p=0.015


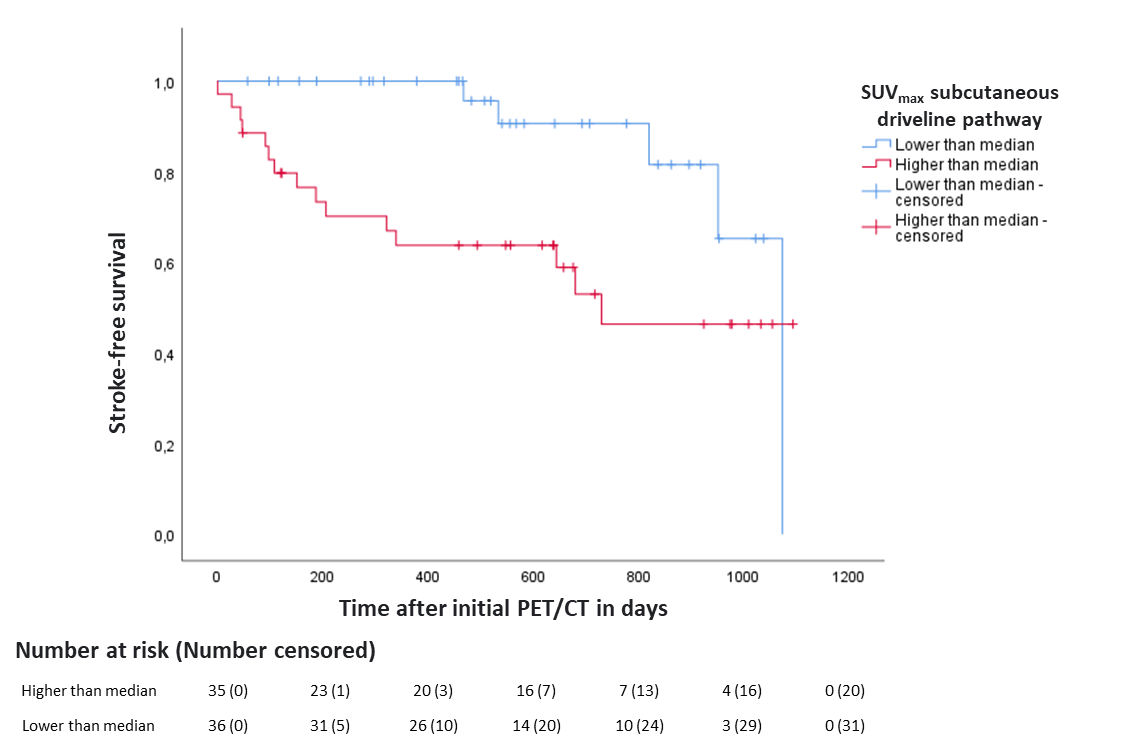


Supplement Figure 3: ROC curve mortality: shows the diagnostic ability of CRP on the morning of the initial PET/CT for prediction of mortality; in four patients CRP was missing; AUC 0.68 (0.58-0.78), p=0.001


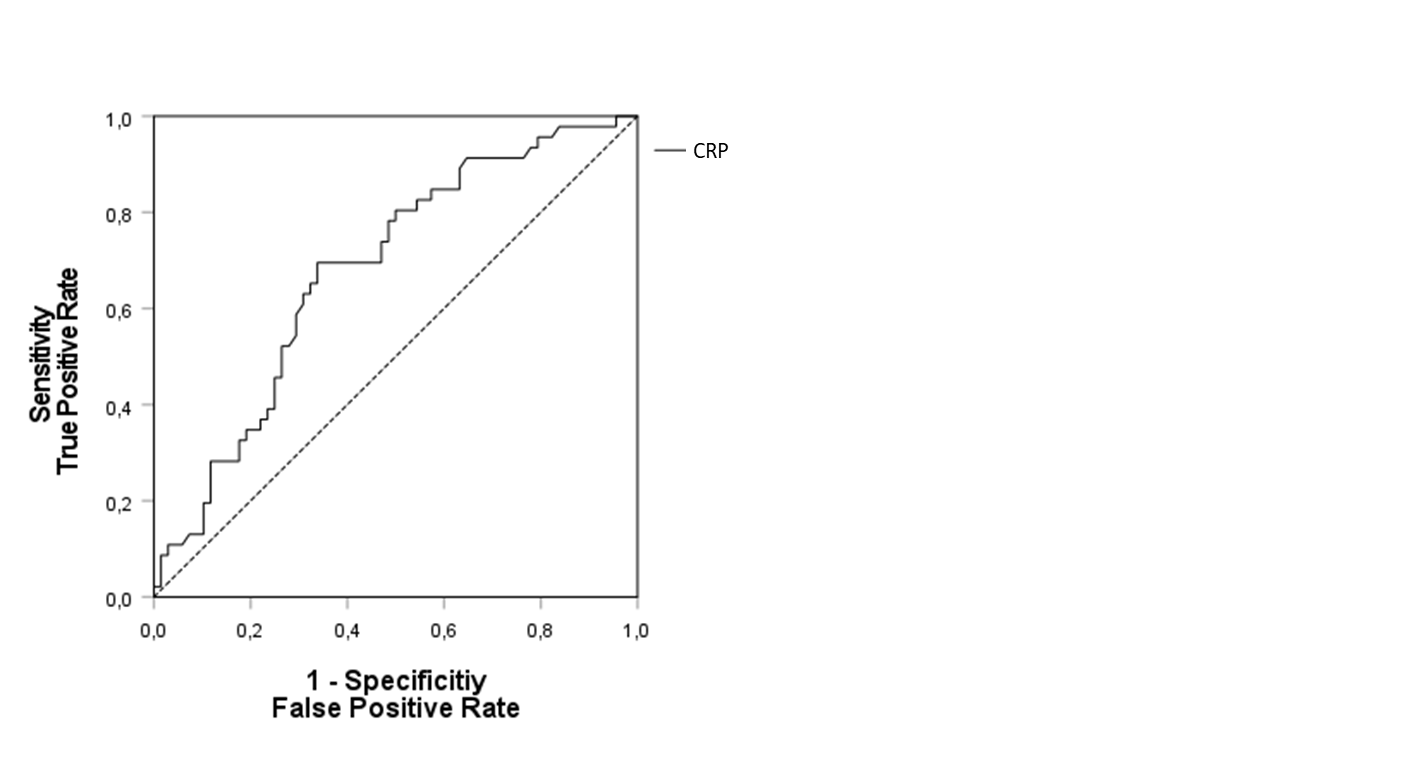


*Supplement Figure 4***:** ROC curve mortality: shows the the diagnostic ability of the activity of the subcutaneous driveline pathway for prediction of mortality; SUV_peak_ subcutaneous driveline pathway: AUC 0.63 (0.53-0.73), p=0.017; SUV_max_ subcutaneous driveline pathway: AUC 0.65 (0.56-0.75), p=0.005; SUV_mean_ subcutaneous driveline pathway: AUC 0.63 (0.53-0.73), p=0.018


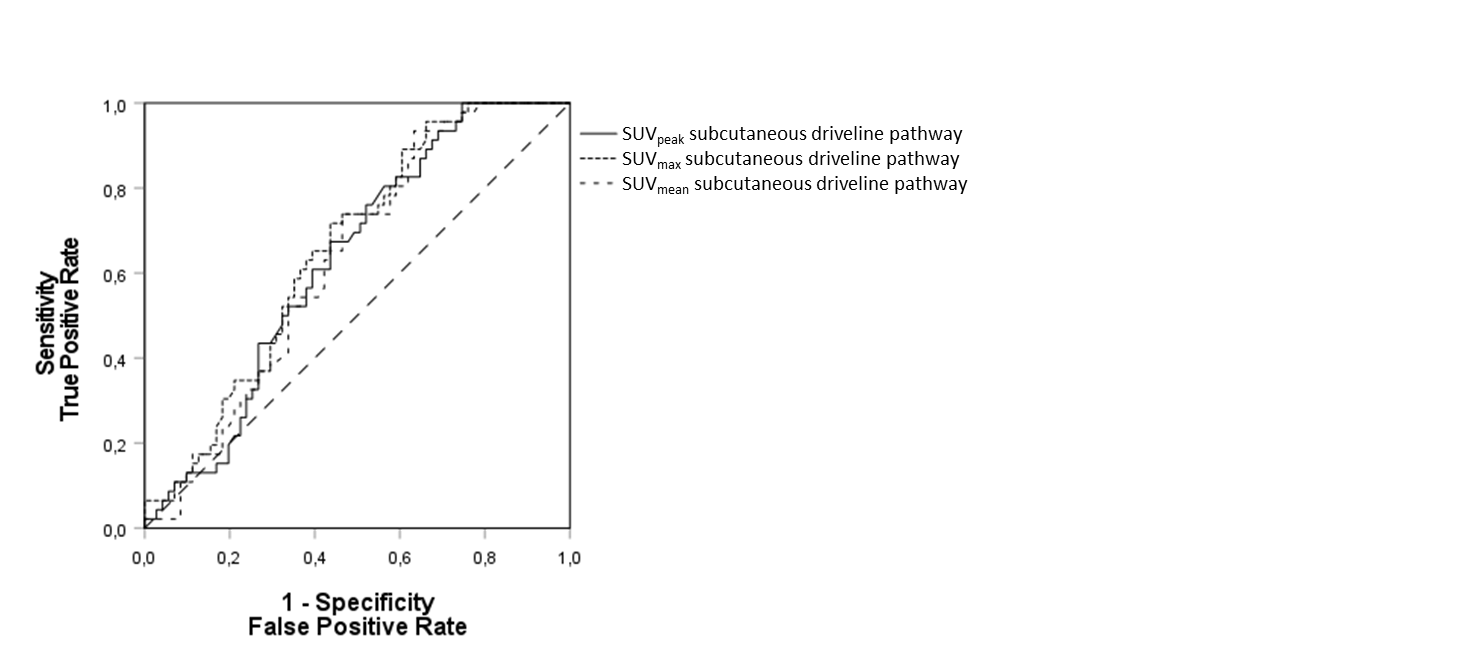

Supplement: Supplementary file 1 — Supplementary Information. [file 41598_2023_45110_MOESM1_ESM.docx]
